# Red meat consumption is associated with prediabetes and diabetes in rural Vietnam: a cross-sectional study

**DOI:** 10.1017/S1368980022001422

**Published:** 2023-05

**Authors:** Chau Que Nguyen, Thuy Thi Phuong Pham, Ami Fukunaga, Dong Van Hoang, Tien Vu Phan, Danh Cong Phan, Dong Van Huynh, Masahiko Hachiya, Huy Xuan Le, Hung Thai Do, Tetsuya Mizoue, Yosuke Inoue

**Affiliations:** 1 Department of Non-communicable Disease Control and Nutrition, Pasteur Institute in Nha Trang, Nha Trang, Khánh Hòa, Vietnam; 2 Department of Epidemiology and Prevention, National Center for Global Health and Medicine, Tokyo 162-8655, Japan; 3 Medical Service Center, Pasteur Institute in Nha Trang, Nha Trang, Khánh Hòa, Vietnam; 4 Khánh Hòa Center for Disease Control, Khánh Hòa, Vietnam; 5 Bureau of International Health Cooperation, National Center for Global Health and Medicine, Tokyo, Japan; 6 Pasteur Institute in Nha Trang, Nha Trang, Khánh Hòa, Vietnam

**Keywords:** Red meat consumption, Processed meat consumption, Poultry meat consumption, Prevalence of diabetes, Prevalence of prediabetes

## Abstract

**Objective:**

To examine the association between red/processed meat consumption and glycaemic conditions (i.e. prediabetes (preDM) and diabetes mellitus (DM)) among middle-aged residents in rural Khánh Hòa, Vietnam.

**Design:**

In this cross-sectional study, a multinomial logistic regression model was used to examine the association between daily consumption of red/processed meat (0–99 g, 100–199 g or ≥ 200 g) and preDM/DM with adjustments for socio-demographic, lifestyle-related and health-related variables.

**Setting:**

Khánh Hòa Province, Vietnam

**Participants:**

The study used data collected through a baseline survey conducted during a prospective cohort study on CVD among 3000 residents, aged 40–60 years, living in rural communes in Khánh Hòa Province.

**Results:**

The multinomial regression model revealed that the relative-risk ratios for DM were 1·00 (reference), 1·11 (95 % CI = 0·75, 1·62) and 1·80 (95 % CI = 1·40, 2·32) from the lowest to the highest red/processed meat consumption categories (*P*_trend_ = 0·006). The corresponding values for preDM were 1·00 (reference), 1·25 (95 % CI = 1·01, 1·54) and 1·67 (95 % CI = 1·20, 2·33) (*P*_trend_ = 0·004). We did not find any evidence of statistical significance in relation to poultry consumption.

**Conclusion:**

Increased red/processed meat consumption, but not poultry consumption, was positively associated with the prevalence of preDM/DM in rural communes in Khánh Hòa Province, Vietnam. Dietary recommendations involving a reduction in red/processed meat consumption should be considered in low- and middle-income countries.

The prevalence of diabetes mellitus (DM) is increasing dramatically and is becoming a health concern, particularly in low- and middle-income countries (LMIC). In 2019, it was estimated that the number of adults with DM in LMIC was approximately 368 million, which is equivalent to four-fifths of the global population with DM; moreover, this number is expected to increase to 588 million by 2045^(1)^. Given that such an increase in the number of individuals with DM could substantially damage population health in LMIC, where preventive measures and proper treatment for DM remain inadequate, additional efforts are required to identify DM risk factors in such countries.

A meta-analysis of fourteen prospective studies conducted across several countries (including the United States, Finland, Japan and China) by Schwingshackl *et al.* ^(2)^ found that the consumption of red and processed meats increased the risk of DM; the pooled risk ratios were 1·17 per 100 g/d of red meat (95 % CI = 1·25, 1·83) and 1·37 per 50 g/d of processed meat (95 % CI = 1·22, 1·55). Previous studies have suggested that the Fe component of haem, which is present in red meat, can result in impaired insulin synthesis and excretion by producing oxidative stress that impairs pancreatic *β*-cells, and increasing Fe storage in the liver which in turn impedes the capacity for insulin extraction^(3–8)^. Other direct and indirect factors have also been suggested, including SFA^(9,10)^; heterocyclic amines and polycyclic aromatic hydrocarbons present in cooked red meat^(11,12)^, excess body weight^(13)^ and metabolic syndrome^(14,15)^. An increasing trend in meat consumption, which has been observed and projected in LMIC^(16)^, might have contributed to the rising rate of DM described above.

The current study was designed to build upon the findings of the aforementioned previous studies in the following manner. First, most studies on meat consumption and DM were from high-income countries^(2)^, and other than data from some studies in China^(17,18)^, there is limited information available from LMIC. For example, no relevant study has been conducted in Vietnam, where the prevalence of DM increased from 2 % to 5 % in men and from 3 % to 5 % in women between 1980 and 2014^(19)^. Second, the association between meat consumption and prediabetes (preDM) has been under-researched. PreDM is an intermediate state of hyperglycaemia with higher-than-normal glycaemic parameters that are still below the threshold for DM^(20)^. Previous studies have linked preDM to a higher risk of DM, CVD, cancer and mortality^(21)^. Given that preDM is a precursor to DM and has a similar pathophysiology, it is possible that haem Fe as well as other pathways (as mentioned above) contribute to the development of preDM as they do in DM. Understanding the association between meat consumption and preDM may facilitate efforts to mitigate the disease burden associated with DM in LMIC.

Therefore, the purpose of the current study was to examine the associations between the consumption of different types of meat (i.e. red/processed meat and poultry) and glycaemic conditions (i.e. preDM and DM) among rural residents in Khánh Hòa Province, Vietnam. We hypothesised that increased red/processed meat consumption, rather than the consumption of poultry (which contains only a small amount of haem), is associated with a higher prevalence of preDM and DM.

## Experimental methods

### Study setting

Data for the current study were obtained through a baseline survey of a prospective cohort study on CVD (i.e. Khánh Hòa Cardiovascular Study). The aim of the Khánh Hòa Cardiovascular Study was to examine the determinants of CVD risk factors among middle-aged residents in rural communes in Vietnam. The study location was purposively selected as it was deemed to be at average affluence in rural Vietnam; Khánh Hòa province ranked 28th out of sixty-three provinces in terms of annual income in 2018; we then chose eight communes in one district in Khánh Hòa. Inclusion criteria included residents who were 40–59 years old at the time of recruitment and who had lived in the current commune for at least 6 months. Based on the member list created by the commune health centre staff, we purposively chose households and invited eligible individuals until the required number of participants was obtained (consent rate, 75–87 %).

As part of the Khánh Hòa Cardiovascular Study baseline survey, we collected anthropometry results, blood samples for biochemical measurements and questionnaire information via face-to-face interviews between June 2019 and June 2020. All the participants were asked to fast for more than 8 h prior to blood collection. Blood specimens were obtained by venepuncture and were centrifuged at the study sites, after which they were transported to the Pasteur Institute in Nha Trang at temperatures below 4°C. Plasma fasting glucose, HDL-cholesterol, LDL-cholesterol and TAG were measured using Cobas 8000 (Roche, Basel, Switzerland), and glycated Hb A1c (HbA1c) was quantified by high-performance liquid chromatography using the HLC-723 G8 system (Tosoh Bioscience, Tokyo, Japan).

### Exposure – Meat consumption

Information on meat consumption was obtained by asking participants questions regarding the frequency and quantity of pork, beef, poultry and sausage/ham consumption. More specifically, we asked them how many days per week they consumed each item. Items that were consumed less than once per week were categorised as ‘none’. For items that were consumed at least once per week, participants were also requested to report the number of times per day they consumed each item and how much of it per occasion. To determine the latter, participants were presented with show-cards depicting pictures of standard portions of each item and were asked to state whether they consumed smaller, equivalent and larger amounts than that shown; these were assigned values of 0·5, 1 and 1·5, respectively. Individuals who consumed more than twice the size of the portion depicted in the show-card were asked to specify that amount using an integer.

We multiplied the above-mentioned values (i.e. consumption frequency and the amount of each food item consumed per occasion (i.e. 100 g of pork and beef, 80 g of poultry and 25 g of ham and sausage based on the portion sizes in the show cards)) to compute the daily meat consumption in grams. Given that the variation in processed meat consumption among the study population was small, we combined red and processed meat. Based on the consumption distribution, we categorised daily meat consumption into the following groups: 0–99 g, 100–199 g and ≥ 200 g for red/processed meat and 0–99 g, 100–199 g, 200–299 g and ≥ 300 g for poultry.

### Outcomes

A participant was diagnosed with DM per the following American Diabetes Association criteria of 2019^(20)^: fasting plasma glucose ≥ 7 mmol/l (≥ 126 mg/dl), HbA1c ≥ 6·5 % or self-reported use of antidiabetic medication. PreDM was defined as an fasting plasma glucose of 5·9–6·9 mmol/l (100–125 mg/dl) or HbA1c of 5·7–6·4 % in individuals without DM^(20)^. Normoglycaemia was defined as an fasting plasma glucose of < 5·6 mmol/l (< 100 mg/dl) and HbA1c of < 5·7 %.

### Covariates

We collected information on socio-demographic covariates including age (in years), sex (man or woman), marital status (married/cohabiting or not married), education (less than primary school, primary school, secondary school or high school or higher), job categories (government employee, non-governmental employee, self-employed, farmer or fisherman, homemaker, other or unemployed) and household income (low, middle or high). Household income was estimated based on the following responses regarding monthly household income: ≤ 1 000 000 Vietnamese đng (VND); 1 000 001–2 000 000 VND; 2 000 001–3 000 000 VND; 3 000 001–4 000 000 VND; 4 000 001–6 000 000 VND; 6 000 001–8 000 000 VND; 8 000 001–12 000 000 VND; 12 000 001–16 000 000 VND; 16 000 001–20 000 000 VND; > 20 000 000 VND or do not know (one USD is equivalent to 23 475 VND as of June 1, 2019). The midpoint of each of these ranges was assigned as a proxy score representing each category. The values were divided by the square root of the number of household members to compute equivalised income, which was then categorised into three groups: low, middle and high. For individuals who were not household representatives, the information collected from the head of household was used as a substitute.

Lifestyle-related variables included smoking status (never, former or current smoker), alcohol consumption (non-drinker, less than 1 standard drink, 1–1·9 standard drinks or ≥ 2 standard drinks), physical activity (wherein participants were divided into tertiles based on the Global Physical Activity Questionnaire^(22)^), daily vegetable consumption (none, 0·1–0·9 cups, 1–1·9 cups, 2–2·9 cups or ≥ 3 cups), fruit consumption (none, 0·1–0·9 cups, 1–1·9 cups or ≥ 2 cups), sweetened beverage consumption (none, 0·1–1·9 cans or ≥ 2 cans) and sleeping hours (the sum of the duration of night sleep and naps: < 6·0, 6·0–6·9, 7·0–7·9, 8·0–8·9 or ≥ 9·0 h).

Height to the nearest 0·1 cm and weight to the nearest 0·1 kg were measured by trained staff members using a portable stadiometer (Charder, HM200P, Tokyo, Japan) and digital scale (Tania, HD-661, Tokyo, Japan), respectively. BMI was calculated by dividing the weight (kg) by the square of the height (m^2^). Blood pressure was measured twice with the participants seated and their arms supported at the heart level using an electronic sphygmomanometer (Omron, HEM1020, Tokyo, Japan). Participants were instructed to rest for at least 5 min prior to the first measurement. The two measurements were used to calculate the mean systolic and diastolic blood pressure. Hypertension was defined as a systolic blood pressure ≥ 140 mmHg, diastolic blood pressure ≥ 90 mmHg or the self-reported use of antihypertensive medication. Dyslipidaemia was defined as LDL-cholesterol ≥ 150 mg/l or HDL-cholesterol < 40 mg/l (men) or < 50 mg/l (women). Family history of DM was assessed by inquiring whether either of the participant’s parents had ever been diagnosed with the condition.

### Statistical analysis

A multinomial logistic regression model was used to examine the association between meat consumption and preDM and DM while accounting for possible heterogeneity at the community level (level 1: individual; level 2: commune). Model 1 was adjusted for age and sex, while model 2 was further adjusted for marital status, education, occupation, household income, smoking status, alcohol consumption, physical activity, sleeping hours, fruit consumption, vegetable consumption, soft drink consumption, BMI, hypertension, dyslipidaemia and family history of DM.

The associations were examined in relation to the consumption of two different meat subtypes (i.e. red/processed meat and poultry). To test the robustness of the study findings, we also conducted a set of sensitivity analyses in which (1) we examined the associations of red meat and processed meat consumption separately and (2) participants who were receiving treatment for DM were excluded.

Results are presented as relative-risk ratios of glycaemic conditions (i.e. preDM and DM) and their corresponding 95 % CI.

## Results

The basic characteristics of the study participants are shown in Table 1; their mean age (sd) was 49·9 (5·5) years. Women accounted for 61·3 % of the participants, and almost 90 % of them were married. Nearly one-fourth (23·9 %) of the participants had at least a high school education. Household socio-economic status was higher among those in the highest meat consumption group. Current smokers accounted for 20·4 % of all participants.

**Table 1:** Basic characteristics of study participants in the Khánh Hòa cardiovascular study, Vietnam (2019–2020)

			Red/processed meat consumption					
	Total participants (*n* 3000)		0–99 g (*n* 2005)		100–199 g (*n* 672)		≥ 200 g (*n* 323)	
	*n*	%	*n*	%	*n*	%	*n*	%
Age								
Mean	49·9		50·1		49·3		49·6	
sd	5·5		5·5		5·6		5·7	
Female	1840	61·3	1378	68·7	346	51·5	116	35·9
Currently married	2691	89·7	1754	87·5	631	93·9	306	94·7
Education								
Less than primary school	352	11·7	285	14·2	43	6·4	24	7·4
Primary school	863	28·8	624	31·1	166	24·7	73	22·6
Secondary school	1068	35·6	689	34·4	252	37·5	127	39·3
High school or higher	717	23·9	407	20·3	211	31·4	99	30·7
Occupation								
Government employee	295	9·8	165	8·2	84	12·5	46	14·2
Non-governmental employee	483	16·1	297	14·8	125	18·6	61	18·9
Self-employed	595	19·8	397	19·8	138	20·5	60	18·6
Farmer/fisherman	870	29·0	592	29·5	186	27·7	92	28·5
Homemaker	527	17·6	414	20·6	82	12·2	31	9·6
Others	111	3·7	68	3·4	27	4·0	16	5·0
Retired/unemployed	119	4·0	72	3·6	30	4·5	17	5·3
Household income								
Low	1002	33·4	739	36·9	183	27·2	80	24·8
Middle	1045	34·8	679	33·9	248	36·9	118	36·5
High	920	30·7	560	27·9	236	35·1	124	38·4
Don’t know	33	1·1	27	1·3	5	0·7	1	0·3
Smoking status								
Never smoker	2036	67·9	1486	74·1	402	59·8	148	45·8
Former smoker	350	11·7	186	9·3	99	14·7	65	20·1
Current smoker	614	20·5	333	16·6	171	25·4	110	34·1
Daily alcohol consumption (standard drinks)								
Non-drinker	2114	70·5	1537	76·7	412	61·3	165	51·1
< 1	416	13·9	241	12·0	121	18·0	54	16·7
1–1·9	201	6·7	92	4·6	64	9·5	45	13·9
≥ 2	269	9·0	135	6·7	75	11·2	59	18·3
Physical activity								
Low	1030	34·3	743	37·1	172	25·6	115	35·6
Middle	975	32·5	662	33·0	235	35·0	78	24·1
High	995	33·2	600	29·9	265	39·4	130	40·2
Sleeping hours per night								
< 6	210	7·0	142	7·1	54	8·0	14	4·3
6–6·9	447	14·9	322	16·1	81	12·1	44	13·6
7–7·9	898	29·9	619	30·9	187	27·8	92	28·5
8–8·9	863	28·8	581	29·0	189	28·1	93	28·8
≥ 9	581	19·4	341	17·0	161	24·0	79	24·5
Missing	1	0·03	0	0·0	0	0·0	1	0·3
Daily fruit consumption (servings)								
0	276	9·2	200	10·0	46	6·8	30	9·3
0·1–0·9	1697	56·6	1151	57·4	365	54·3	181	56·0
1–1·9	670	22·3	436	21·7	165	24·6	69	21·4
≥ 2	357	11·9	218	10·9	96	14·3	43	13·3
Daily vegetable consumption (servings)								
< 1	627	20·9	426	20·9	131	19·5	70	21·7
1–1·9	1284	42·8	932	46·5	241	35·9	111	34·4
2–2·9	705	23·5	416	20·7	205	30·5	84	26·0
≥ 3	384	12·8	231	11·5	95	14·1	58	18·0
Daily standard soft drink consumption (standard drinks)								
0	2546	84·9	1737	86·6	560	83·3	249	77·1
0·1–1·9	189	6·3	115	5·7	53	7·9	21	6·5
2–3·9	138	4·6	82	4·1	27	4·0	29	9·0
≥ 4	127	4·2	71	3·5	32	4·8	24	7·4
BMI								
Mean	23·2		23·3		23·1		23·1	
sd	3·0		3·0		2·8		3·0	
Hypertension	1189	39·6	796	39·7	265	39·4	128	39·6
Dyslipidaemia	1352	45·1	903	45·0	298	44·3	151	46·7
Family history of diabetes mellitus	373	12·4	251	12·5	84	12·5	38	11·8

Table 2 shows results of multinomial logistic regression model examining the association between meat consumption and glycaemic conditions. We found that red/processed meat consumption was significantly associated with both DM and preDM; the relative-risk ratios for the highest consumption category (i.e. ≥ 200 g) were 1·55 (95 % CI = 1·14, 2·11) for DM and 1·58 (95 % CI = 1·20, 2·09) for preDM. These associations remained statistically significant after adjusting for several covariates (relative-risk ratios = 1·80 (95 % CI = 1·40, 2·32) and 1·67 (95 % CI = 1·20, 2·33), respectively). We found no significant associations between poultry consumption and either DM or preDM.

**Table 2:** Results of multinomial logistic regression analyses of the association between meat consumption and prediabetes or diabetes among community-dwelling adults in rural Khánh Hòa province, Vietnam (2019–2020)

	*n*	Prediabetes					Diabetes mellitus				
		*n*	Model 1		Model 2		n	Model 1		Model 2	
			Relative-risk ratios	95 % CI	Relative-risk ratios	95 % CI		Relative-risk ratios	95 % CI	Relative-risk ratios	95 % CI
1. Red/processed meat consumption											
0–99 g	2005	905	1·00 (Ref.)		1·00 (Ref.)		209	1·00 (Ref.)		1·00 (Ref.)	
100–199 g	672	323	1·18	0·98, 1·42	1·25	1·01, 1·54	61	1·01	0·67, 1·53	1·11	0·75, 1·62
≥ 200 g	323	173	1·58	1·20, 2·09	1·67	1·20, 2·33	37	1·55	1·14, 2·11	1·80	1·40, 2·32
			*P*_trend_ = 0·002		*P*_trend_ = 0·004			*P*_trend_ = 0·124		*P*_trend_ = 0·006	
2. Poultry consumption											
0–99 g	1500	731	1·00 (Ref.)		1·00 (Ref.)		157	1·00 (Ref.)		1·00 (Ref.)	
100–199 g	1130	495	0·84	0·66, 1·07	0·85	0·70, 1·05	118	0·97	0·77, 1·22	1·02	0·79, 1·32
200–299 g	209	100	0·92	0·70, 1·20	0·92	0·76, 1·11	18	0·84	0·50, 1·41	0·76	0·53, 1·11
≥ 300 g	161	75	0·87	0·57, 1·32	0·84	0·57, 1·23	14	0·81	0·36, 1·82	0·71	0·30, 1·69
			*P*_trend_ = 0·283		*P*_trend_ = 0·179			*P*_trend_ = 0·506		*P*_trend_ = 0·399	

The results are shown as relative-risk ratios and corresponding 95 % CI.

Model 1 was adjusted for age, age squared term and sex, while model 2 was further adjusted for other socio-demographic variables (education, occupation and household income), lifestyle variables (smoking status, alcohol consumption, physical activity, sleeping hours, fruit consumption, vegetable consumption and sweetened beverage consumption) and health-related variables (BMI, hypertension, dyslipidaemia and family history of diabetes). When the consumption of red/processed meat and poultry was examined, the exposures were simultaneously incorporated into the models.

Separate analyses of red meat and processed meat consumption revealed DM/preDM to be significantly associated with red meat consumption but not with processed meat consumption (online Supplementary Table 1). There remained no significant association between poultry consumption and DM/preDM. Sensitivity analysis in which we excluded patients receiving antidiabetic treatment also revealed that red/processed meat consumption, but not poultry consumption, was significantly associated with a higher prevalence of preDM and DM (Table 3).

**Table 3:** Results of multinomial logistic regression analyses of the association between meat consumption and prediabetes or diabetes among community-dwelling adults in rural Khánh Hòa province, Vietnam (2019–2020), excluding participants receiving treatment for diabetes medication (*n* 2907)

		Prediabetes					Diabetes mellitus				
			Model 1		Model 2			Model 1		Model 2	
	*n*	*n*	Relative-risk ratios	95 % CI	Relative-risk ratios	95 % CI	*n*	Relative-risk ratios	95 % CI	Relative-risk ratios	95 % CI
1. Red/processed meat consumption											
0–99 g	1944	905	1·00 (Ref.)		1·00 (Ref.)		148	1·00 (Ref.)		1·00 (Ref.)	
100–199 g	651	323	1·18	0·98, 1·42	1·25	1·02, 1·55	40	0·93	0·53, 1·62	1·06	0·64, 1·74
≥ 200 g	312	173	1·58	1·19, 2·09	1·66	1·19, 2·33	26	1·53	1·05, 2·22	1·75	1·32, 2·33
			*P*_trend_ = 0·003		*P*_trend_ = 0·004			*P*_trend_ = 0·329		*P*_trend_ = 0·045	
2. Poultry consumption											
0–99 g	1447	731	1·00 (Ref.)		1·00 (Ref.)		104	1·00 (Ref.)		1·00 (Ref.)	
100–199 g	1099	495	0·84	0·66, 1·07	0·86	0·70, 1·05	87	1·08	0·90, 1·29	1·12	0·90, 1·40
200–299 g	202	100	0·92	0·71, 1·20	0·92	0·77, 1·10	11	0·77	0·46, 1·31	0·71	0·50, 1·01
≥ 300 g	159	75	0·87	0·58, 1·32	0·84	0·57, 1·22	12	1·06	0·48, 2·37	0·88	0·39, 2·01
			*P*_trend_ = 0·294		*P*_trend_ = 0·183			*P*_trend_ = 0·989		*P*_trend_ = 0·669	

The results are shown as relative-risk ratios and corresponding 95 % CI.

Model 1 was adjusted for age, age squared term and sex, while model 2 was further adjusted for other socio-demographic variables (education, occupation and household income), lifestyle variables (smoking status, alcohol consumption, physical activity, sleeping hours, fruit consumption, vegetable consumption and sweetened beverage consumption) and health-related variables (BMI, hypertension, dyslipidaemia and family history of diabetes). When the consumption of red/processed meat and poultry was examined, the exposures were simultaneously incorporated into the models.

## Discussion

Among 3000 middle-aged community dwellers in rural Khánh Hòa, Vietnam, we found that individuals who consumed ≥ 200 g/d of red/processed meat were 1·67 times and 1·80 times more likely to have preDM and DM, respectively, than in those who consumed < 100 g/d. We found no evidence of a significant association between poultry consumption and preDM or DM.

The significant association between red and processed meat consumption and DM as revealed in our study is consistent with the findings of previous studies^(2,23,24)^. In particular, our results are consistent with data from studies in Asia that examined the association between red meat consumption and DM^(17,18,25)^, including Du *et al.*’s study of individuals registered in the China Kadoorie Biobank^(17)^ (hazard ratio = 1·11, 95 % CI = 1·04, 1·20 per 50 g increase) and Kurotani *et al.*’s study of a Japanese population^(25)^ (OR = 1·48, 95 % CI = 1·15, 1·90 for the highest *v*. lowest quartile).

Notably, this significant association between red/processed meat consumption and DM has also been reported in other LMIC. In addition to Du *et al.*’s aforementioned study^(17)^, another investigation in Brazil found that red meat consumption was associated with a 40 % increase in the risk of DM (95 % CI = 1·04, 1·96) among men^(26)^. Even though red meat (particularly beef) is rich in oleic acid, which reportedly plays a role in countering insulin resistance^(27)^, our findings and those of others suggest that the increase in red meat consumption in LMIC may nevertheless be contributing to the rising prevalence of DM in these countries, where people have been experiencing rapid economic growth.

Our finding that there was no significant association between poultry consumption and DM was consistent with findings reported in an umbrella review by Neuenschwander *et al.* ^(28)^, who reported no significant association (hazard ratio = 1·05 per 100 g/d of poultry consumption, 95 % CI = 0·91, 1·22). Our results also agreed with those of Du *et al.* and Kurotani *et al.*’s studies of Asian populations that found no association between poultry consumption and DM^(17,25)^. It should be also of note that a study of 74 493 middle-aged women in China by Villegas *et al.* even found an inverse association between poultry consumption and DM^(18)^. The apparent non-involvement of poultry consumption in DM development may be attributable to its lower levels of haem (the Fe component of which can impair insulin synthesis and excretion) compared with red meat.

To our knowledge, no studies have specifically examined the association between meat consumption and preDM, irrespective of the study location. Despite a slightly different exposure, however, Cao *et al.* ^(29)^ found a significant association between a dietary pattern characterised by high meat consumption (without distinguishing the types of meat) and preDM among 7555 urban and rural residents in Jiangsu, China (OR = 1·229, 95 % CI = 1·077, 1·402). Identifying individuals at a risk of developing DM at an earlier stage (i.e. before any clinical manifestations) may help mitigate the disease burden associated with DM. This is particularly important in LMIC, where medical and economic resources are insufficient and where the disease burden associated with diabetes is expected to increase due to population ageing, urbanisation and economic growth^(1)^.

The current study had several limitations. First, we used show-cards to determine the amount of meat consumption; this method has not been validated and is therefore potentially subject to measurement error. Second, we did not collect information on the consumption of different types of red meat (i.e. processed *v*. unprocessed red meat), which has been suggested to have different health implications^(25,30,31)^. Third, owing to the cross-sectional design of the study, we were unable to infer causality (e.g. those diagnosed with DM may have altered their meat consumption habits as part of their efforts to delay or prevent DM-related complications). Fourth, our study participants might not be representative of the Vietnamese population as a whole given that they were middle aged and residing in rural communes within a single province. Thus, caution should be exercised when generalising the study findings to other populations.

## Conclusion

Our study of 3000 rural residents of Khánh Hòa Province, Vietnam, revealed that the consumption of red/processed meat consumption, but not poultry consumption, was associated with the increased prevalence of both DM and preDM. Dietary recommendations that involve a reduction in red/processed meat consumption should be considered in LMIC, where there has been an increasing trend in meat consumption during the course of economic growth in the past few decades.

## Supplementary material

For supplementary material accompanying this paper visit https://doi.org/10.1017/S1368980022001422. click here to view supplementary material
