# Supplementary material for: Red meat consumption is associated with prediabetes and diabetes in rural Vietnam: a cross-sectional study
Source: Public Health Nutr. 2022 Jun 20;26(5):1006–13. doi: 10.1017/S1368980022001422 (PMC10346020; doi:10.1017/S1368980022001422)
Supplement: Supplementary file 1 [file S1368980022001422sup001.docx]

**Supplementary Table 1.** Results of multinomial logistic regression analyses of the association between meat consumption and prediabetes or diabetes among community-dwelling adults in rural Khanh Hoa Province, Vietnam (2019-2020), with red meat and processed meat separately examined

|  | N | Prediabetes | | |  | Diabetes mellitus | | |
| --- | --- | --- | --- | --- | --- | --- | --- | --- |
|  |  | n | Model 1 | Model 2 |  | n | Model 1 | Model 2 |
| Red meat consumption | | | | | | | | |
| 0 – 99 g | 2007 | 906 | 1.00 (Ref.) | 1.00 (Ref.) |  | 209 | 1.00 (Ref.) | 1.00 (Ref.) |
| 100 – 199 g | 670 | 322 | 1.18 (0.98-1.41) | 1.25 (1.01-1.53) |  | 61 | 1.02 (0.66-1.55) | 1.12 (0.75-1.66) |
| ≥200 g | 323 | 173 | 1.58 (1.20-2.09) | 1.67 (1.20-2.33) |  | 37 | 1.56 (1.15-2.11) | 1.80 (1.41-2.31) |
|  |  |  | p _trend_ = 0.002 | p _trend_ = 0.003 |  |  | p _trend_ = 0.119 | p _trend_ = 0.005 |
| Processed meat consumption |  |  |  |  |  |  |  |  |
| No | 2956 | 1378 | 1.00 (Ref.) | 1.00 (Ref.) |  | 304 | 1.00 (Ref.) | 1.00 (Ref.) |
| Yes | 44 | 23 | 1.31 (0.88-1.96) | 1.33 (0.77-2.30) |  | 3 | 0.76 (0.16-3.69) | 0.66 (0.09-4.98) |
|  |  |  |  |  |  |  |  |  |
| Poultry consumption |  |  |  |  |  |  |  |  |
| 0 – 99 g | 1500 | 731 | 1.00 (Ref.) | 1.00 (Ref.) |  | 157 | 1.00 (Ref.) | 1.00 (Ref.) |
| 100 – 199 g | 1130 | 495 | 0.83 (0.65-1.06) | 0.85 (0.70-1.04) |  | 118 | 0.97 (0.78-1.22) | 1.03 (0.79-1.33) |
| 200 – 299 g | 209 | 100 | 0.92 (0.70-1.20) | 0.91 (0.75-1.11) |  | 18 | 0.84 (0.50-1.41) | 0.77 (0.53-1.10) |
| ≥300 g | 161 | 75 | 0.87 (0.57-1.33) | 0.84 (0.57-1.23) |  | 14 | 0.81 (0.36-1.82) | 0.70 (0.30-1.66) |
|  |  |  | p _trend_ = 0.277 | p _trend_ = 0.175 |  |  | p _trend_ = 0.505 | p _trend_ = 0.399 |

The results are shown as relative-risk ratios and corresponding 95% confidence intervals.

Model 1 was adjusted for age, age squared term, and sex, while Model 2 was further adjusted for other sociodemographic variables (education, occupation, and household income), lifestyle variables (smoking status, alcohol consumption, physical activity, sleeping hours, fruit consumption, vegetable consumption, and sweetened beverage consumption), and health-related variables (body mass index, hypertension, dyslipidemia, and family history of diabetes). When the consumption of red meat, processed meat, and poultry was examined, the exposures were simultaneously incorporated into the models.
